# Genetic and Functional Profiling of Crohn's Disease: Autophagy Mechanism and Susceptibility to Infectious Diseases

**DOI:** 10.1155/2013/297501

**Published:** 2013-05-08

**Authors:** Annalisa Marcuzzi, Anna Monica Bianco, Martina Girardelli, Alberto Tommasini, Stefano Martelossi, Lorenzo Monasta, Sergio Crovella

**Affiliations:** ^1^Institute for Maternal and Child Health—IRCCS “Burlo Garofolo” of Trieste, Via dell'Istria 65/1, 34137 Trieste, Italy; ^2^University of Trieste, 34127 Trieste, Italy

## Abstract

Crohn's disease is a complex disease in which genome, microbiome, and environment interact to produce the immunological background of the disease. Disease in childhood is more extensive and characterized by a rapid progression, leading to severe repercussions in the course of the disorder. Several genetic variations have been associated with an increased risk of developing the disease and most of these are also implicated in other autoimmune disorders. The gut has many tiers of defense against incursion by luminal microbes, including the epithelial barrier and the innate and adaptive immune responses. Moreover, recent evidence shows that bacterial and viral infections, as well as inflammasome genes and genes involved in the autophagy process, are implicated in Crohn's disease pathogenesis. The aim of this review is to establish how much the diagnostic system can improve, thus increasing the success of Crohn's disease diagnosis. The major expectation for the near future is to be able to anticipate the possible consequences of the disease already in childhood, thus preventing associated complications, and to choose the best treatment for each patient.

## 1. Introduction 

Crohn's disease (CD) is a chronic form of inflammatory bowel disease (IBD) that can affect any part of the gastrointestinal tract, from the mouth to the anus. However, it most commonly affects the colon and terminal ileum [1] with up to 75% of patients having ileal disease with or without colonic involvement [2]. It is a debilitating disorder with an overall prevalence of 0.5%–1% of the general population [3, 4]. CD differs from other types of IBDs because in patients with CD the inflammation is often continuous and with involvement of the mucosa [5]. 

Complications are common but not a constant: disease progression is marked by severe colitis, strictures and perianal fistulas, typically requiring surgery [6, 7]. 

Beaugerie et al. [8] recently reported three factors that at the time of the diagnosis increase the chance of developing a disabling disease in the following five years: (A) age <40 years, (B) presence of perianal lesions, and (C) the requirement of steroids to control the first flare [8]. However, as the median age at diagnosis is 27 years, patients may live with CD for more than 50 years in the Western world, where life expectancy of patients exceeds 70 years.

The age of onset is frequently in the second decade of life, and most patients progress to a relapsing disease characterized by abdominal pain, bloody diarrhea, vomit, and weight loss. 

Although CD normally manifests in adulthood, it can be present in childhood before the age of 2 years [9]. The early onset Crohn's disease (EOCD) is typically more extensive (beyond the colon and/or oral or perianal disease) and characterized by rapid progression, leading to severe repercussions in disease development [10]. Diagnosis is particularly challenging in children in which presenting symptoms may vary widely and may only consist of subtle extraintestinal manifestations [11]. This often leads to a typical delay in the diagnosis of pediatric IBD, ranging from 4 weeks in severe colitis to 6-7 months in milder disease. Reducing this diagnostic delay is important, since a long period of unmanaged symptoms can significantly impact on growth and early treatment is essential to preserve long-term quality of life [12, 13]. Thus, a sensitive yet noninvasive tool for the identification of patients at high risk of IBD, and therefore warranting endoscopic evaluation, would be a valuable diagnostic aid. There are specific clinical, therapeutic, and psychosocial issues specific to children with IBD that must be considered to ensure prompt diagnosis and appropriate medical management.

The etiopathogenesis of CD is unclear. It remains to be determined whether this disease represents an abnormal response to normal antigenic stimuli or an appropriate response to persistently abnormal stimuli [14, 15]. A better understanding of the origin of the disease and the mechanisms of action is necessary to improve the prognosis of CD. 

CD is a complex disorder resulting from the interaction of genetic environmental and microbial factors. Given the difficult genotype-phenotype correlation and given the heterogeneous genetics, the different interactions among predisposing factors, and not only the number of genes involved, should be considered in order to understand the mechanisms of this disease. Many previous and ongoing studies have sought to identify genes and especially disease-causing variants such as risk factors for the disease. Identification and characterization of disease-causing variants represents one of the biggest challenges of genetics within the etiopathogenetic study of CD. Genomewide association studies already identified several distinct genetic polymorphisms associated with Crohn's disease. In European individuals, NOD2 gene polymorphisms confer by far the greatest risk for the disease. Other variations in ATG16L1, IRGM, and IL23R genes were reported to be highly associated with CD. 

The genetic background has certainly a predisposing role, but several alternative explanations are possible, mostly related to lifestyle. The importance of environmental factors is shown by an increase in the incidence rate of diseases in ethnic groups previously less affected, as Hispanic, Asians, and immigrants that had moved from regions of low incidence into areas where the incidence of the disease is higher [16]. 

Observation of Crohn's patients and animal models suggests a role of bacteria in the disorder [17]. Among the most important bacteria that can adhere and invade the mucosa is *Escherichia coli *[18]. The onset of the disease is quite common after gastrointestinal infections and people suffering from this disorder have, generally, higher concentrations of mucosal bacteria if compared to healthy subjects [19]. 

A detailed picture of how genes work together and interact with environmental and microbial factors may better explain individual differences in CD susceptibility. 

## 2. Etiology of CD

The complex pathophysiology of CD has, for a long time, been an enigma [20]. 

**Figure 1:**
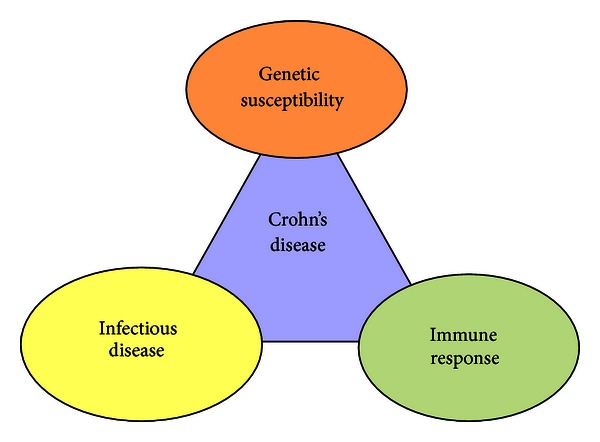
Crohn's disease is a heterogeneous disorder of multifactorial etiology in which genetic, environmental, and microbial factors, together with the immunological response, interact to produce the disease.

Although the precise etiology of CD remains elusive, epidemiological data conclusively indicate a deregulation of the immune response against the luminal flora in a genetically susceptible host [21] (Figure 1). 

It is commonly assumed that CD is a heterogeneous disorder of multifactorial etiology in which genome, microbiome (hereditability), and environment interact to produce the immunological background of the disease. It is probable that patients have a genetic predisposition for the development of the disease coupled with immunoregulation disturbances [22]. 

### 2.1. Genetic Susceptibility: Greater Weighing Factor in Early Onset Crohn Disease

**Figure 2:**
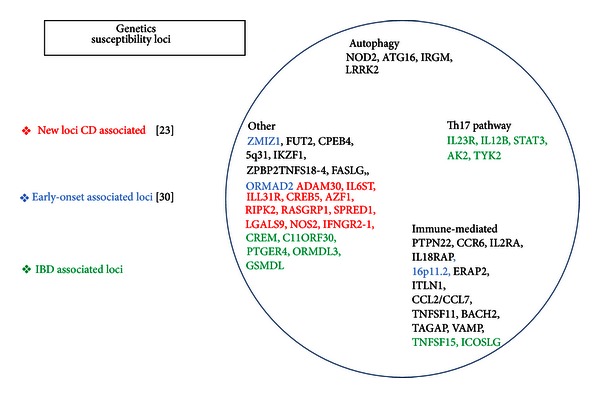
More than 90 distinct genomic susceptibility loci have been found to be associated with an increased risk of developing CD. The genes variants relate largely to the innate immunity genes, in particular to the disruption of the innate and adaptative arms of the immune systems, to the process of autophagy, to the epithelial barrier function, and to the activation of the endoplasmic reticulum stress response.

The epidemiologic evidence of the role of genetic factors in the pathogenesis of the disease came from studies demonstrating higher rates of CD among individuals of Caucasian and Jewish ethnicity, familial aggregation of CD, and higher concordance rates of both twins developing CD in monozygotic compared to dizygotic twins. Due to the complexity of the disease, the search for specific CD susceptibility genes has been very difficult so far. Despite the large number of genomewide associations (GWAS) established to date, most complex diseases (not monogenic) have only managed to explain some additional percentage of the hereditability estimates. The source of this *missing hereditability* is the subject of much debate with various explanations: overestimates of original heritability statistics, underpowered GWAS studies to detect common variants, poorly investigated epistasis and gene-environment interactions, and rare genetic variants [23]. In the attempt to explain some of this *missing hereditability*, researchers have adopted several complementary strategies. Combined genotypes, “private genes,” and epigenetic markers may account for this *missing hereditability*: monogenic immunity disorders are increasingly diagnosed in patients with EOCD [24]. Advances in bioinformatics have now made it possible to perform GWAS using copy number variation probes. By several GWAS and meta-analysis studies many genes have been associated with CD: more than 90 distinct genomic loci have been found to be associated with an increased risk of developing CD. Genes with replicated evidence for strong association suggest that these variants relate largely to the innate immunity genes, in particular to the disruption of the innate and adaptive arms of the immune system, to the process of autophagy, to the epithelial barrier function, and to the activation of the endoplasmic reticulum stress response [25–27] (Figure 2). 

Most loci are in relation with the CD phenotype and many loci are implicated in other immune-mediated disorders, most notably with ankylosing spondylitis, erythema nodosum, and psoriasis [28]. Several genes are involved in primary immunodeficiencies, characterized by a dysfunctional immune system resulting in severe infections [26, 29, 30]. In the last years defective processing of intracellular bacteria has become a central theme. A considerable overlap has also been observed between susceptibility loci for CD and susceptibility for infectious diseases [27]. 

Genetic susceptibility is thought to play a more important role in the etiology of early rather than late onset CD [31]. This is supported by a higher rate of positive family history of CD in patients with a younger age at diagnosis with respect to patients with older age at diagnosis, suggesting that an earlier presentation may be due to a higher burden of disease-causing mutations in the genomes of these affected children compared to those in whom disease manifests later in life [32]. EOCD presents a more aggressive phenotype; in fact earlier age at diagnosis is associated with a greater need for surgery [33]. EOCD candidate susceptibility genes have been identified using linkage analysis and gene sequencing in two unrelated consanguineous families [34]. Some gene/loci may be specific to pediatric-onset CD and that is documented by recent GWAS focused on a pediatric cohort highlighting the implication of novel pathways and interaction between the two different onsets [35, 36].

Moreover, environmental factors such as smoking are less likely to be exerting an influence on the disease in pediatric cohorts [37]. 

### 2.2. Gastrointestinal Microbiota: Host Genome-Microbe Interactions in Crohn's Pathogenesis

Considering epidemiological, genetic and immunological data, it is probable that patients have a genetic predisposition for the development of the disease coupled with disturbances in both immunoregulation, and intestinal microbiota. The disease can be triggered by any of a number of different factors and sustained by an abnormal immune response to these factors. Rather, the intensive interaction between intestinal epithelial cells and immune competent cells is critical to maintain and perpetuate the chronic inflammatory process characteristic of CD [22]. The genetic and pathological complexity of CD is particularly well suited for testing whether interactively redefining disease diagnoses can enhance the value of genetic and pathogenetic studies. Precision in the characterization of the disease would make defining the impact of host-gene-microbial interactions on the disease process more robust. 

The human body is inhabited by a vast number of bacteria, archaea, viruses, and unicellular eukaryotes. The microbiota represents a collection of microorganisms that live in peaceful coexistence with their hosts [38]. By far, the most heavily colonized organ is the gastrointestinal tract (GIT) and the colon alone is estimated to contain over 70% of all the microbes [39] and represents a major surface for microbial colonization. It is estimated that the human microbiota, not homogeneous in the GIT, contains as many as 1014 bacterial cells [40, 41] and the number of bacterial species present in the human gut is estimated to be 500 to 1000 [42]. Nevertheless, a recent analysis involving multiple subjects has suggested that the collective human gut microbiota is composed of over 35000 bacterial species [43]. 

**Figure 3:**
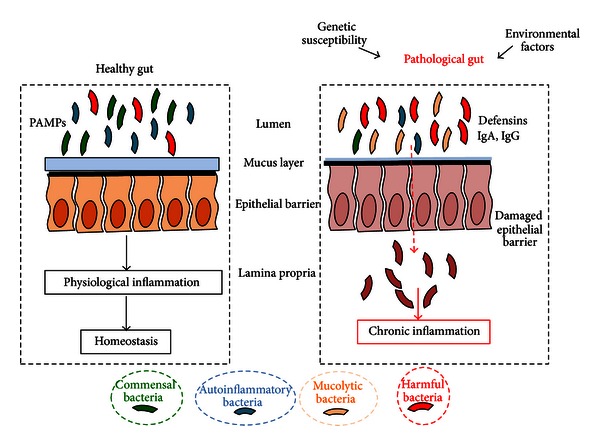
The role of microbiota in Crohn's disease pathogenesis. The interplay between the host microbiota and the environmental factors in a genetic susceptible host results in a progressive inflammatory damage to the host intestinal mucosa.

Colonization of the human gut with microbes begins immediately at birth; in fact infants are exposed to a complex microbial population upon the passage through the birth canal [44] and it is known that infants delivered through cesarean section have different microbial compositions compared to vaginally delivered infants [45]. It has been shown that the microbiota of adult monozygotic and dizygotic twins was equally similar to that of their siblings, suggesting that the colonization by the microbiota from a shared mother was more decisive in determining their adult microbiota than their genetic makeup [46] (Figure 3).

Several studies have shown that host genetics can impact the microbial composition of the gut [47, 48]. The central role of gut microbiota in the development of mucosal immunity is not surprising considering that the intestinal mucosa represents the largest surface area in contact with the antigens of the external environment called PAMPs (pathogen-associated molecular patterns). Additionally, the dense carpet of the gut microbiota overlying the mucosa normally accounts for the largest proportion of the antigens presented to the resident immune cells and those stimulating the pattern recognition receptors such as the NOD-like receptors (NLRs) of the intestinal epithelial cells [49]. The GI (gastrointestinal gut) microbiome of healthy humans is dominated by four major bacterial phyla: *Firmicutes*, *Bacteroidetes*, and to a lesser degree *Proteobacteria* and *Actinobacteria* [50]. Many studies have observed imbalances or dysbioses in the GI microbiomes of CD patients [51, 52]. In CD patients biodiversity is decreased, with a lower proportion of *Firmicutes* and an increase in Gammaproteobacteria [53]. In CD, proportions of the Clostridia are altered: the *Roseburia* and *Faecalibacterium* genera of the *Lachnospiraceae* and *Ruminococcaceae* families are decreased, whereas *Ruminococcus gnavus* increases [54]. An important concept in the pathogenesis of CD is that bacterial and viral interactions occur in a host gene-specific manner [55, 56]. Surgical diversion of the fecal stream ameliorates inflammation. In addition, the evaluation of the microbial populations in surgically resected tissue samples of small bowel and colon from CD patients and non-CD controls, by rRNA sequence analysis, showed that specific flora was not enriched in small bowel or colon from CD patients. However, a subset of CD samples showed alterations in the representations of the *Bacteroides* and *Firmicutes* [43, 57]. Moreover, several studies have shown that the gut microbiota is altered in IBD patients. For example, biopsy samples from CD patients were used to prepare bacterial DNA which was amplified using universal bacterial 16S rRNA primers [58], and a significant increase in *Proteobacteria* and *Bacteroidetes* was found in CD patients compared to controls, with a decrease in *Clostridia*. Metagenomic approaches were used to analyze fecal samples from Crohn's patients and healthy donors and revealed reduced complexity of the *Firmicutes* in affected individuals [59]. Finally, the evidence that intestinal bacteria play an important role in CD patients is that antibiotics help some patients and can ameliorate disease activity. Moreover metronidazole is an important therapeutic agent for certain complications of CD such as fistulising disease. Viral infection is required to generate the Paneth cell defect found in ATG16L1 mice [60], suggesting that in addition to human bacterial microbiota, viral or fungal commensals may play a role in CD pathogenesis.

### 2.3. Immunological Response in CD

#### 2.3.1. Th1 and Th17 Implicated in CD Pathogenesis

Although the exact CD etiology is still not completely understood, several studies indicate that its pathogenesis is characterized by an exaggerated immune response in genetically susceptible individuals. 

**Figure 4:**
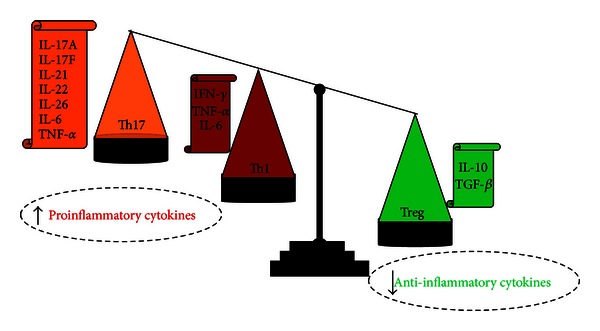
Up- and Downregulation of the proinflammatory cytokines evidenced in the immune system dysregulation of CD patients.

CD patients suffer from marked immune system deregulation. The inflammation seen in these patients is characterized by pronounced Th1 and Th17 responses [61] involving upregulation of proinflammatory cytokines IL-1, IL-6, IL-12, TNF-*α*, IFN-*γ*, IL-23, and IL-17 and downregulation of IL- 10, but it is not clear whether this is a cause or a consequence of the disease [62] (Figure 4). 

Th1 cells are commonly assumed to be associated with CD development and produce IFN- *γ*, and their primary role is the protection against intracellular microbes. IFN-*γ* secreting lamina propria lymphocytes are abundant in the mucosa of CD patients: this condition is marked at CD onset (mucosal T cells appear to mount a typical Th1 response that resembles an acute infectious process) and disappears in late CD. 

Recently, several studies showed the pivotal role of the imbalance of regulatory T cells (Treg) and Th17 in CD. Treg cells are important for the control of the immune response to self-antigens preventing autoimmunity and maintaining self-tolerance [63]. In contrast, IL-17 producing Th17 cells were recognized as a novel group of T cells which play a major role in autoimmunity. The gastrointestinal immune system has to maintain both a state of tolerance toward intestinal antigens and the ability to combat pathogens. In CD this balance is lost and the effects of proinflammatory T cells outnumber the tolerizing, anti-inflammatory effects of Treg cells. The discovery that Th17 cells, which express the IL-23 receptor (IL-23R), play a role in CD pathogenesis was supported by recent GWAS studies demonstrating that IL-23R and other genes involved in the differentiation of Th17 cells are susceptibility genes. 

To confirm the link between immune response and genetic susceptibility in the pathogenesis of CD there are several recent lines of evidence that the key role is played by autophagy that includes the antigen presentation and the production of proinflammatory cytokines. The relationship between autophagy and microbes, indeed, has remained ill-defined until a recent convergence of studies showing that autophagy is an innate immune defense against bacteria, protozoa, and viral pathogens [64]. It is commonly assumed that the role of autophagy in addition to eliminating intracellular pathogens [65] contributes to MHC II restricted endogenous antigen presentation. It is an effector of Th1/Th2 polarization, affects B and T cell homeostasis and repertoire selection, delivers cytosolic PAMP or danger associated molecular patterns to endosomal toll-like receptors (TLR), and acts as an innate immunity effector downstream of TLR [66]. Polymorphisms in autophagy genes result in deregulation of these processes and affect gut homeostasis: genetic variants of autophagy genes have been linked to CD. 

#### 2.3.2. The Role of the NLRP3 Inflammasome in the Pathogenesis of CD

Inflammasomes are cytoplasmic multiprotein complexes that function as sensors of endogenous or exogenous PAMPs. They are composed of one of several nucleotide-binding oligomerization-domain protein-like receptors (NLRs), including NLRP1, NLRP3, NLRP6, and NLRPC4. Upon sensing the relevant signal, they assemble, typically together with an adaptor protein, an apoptosis-associated speck-like protein (ASC) or a caspase activating and recruitment domain 8 (CARD8), into a multiprotein complex that governs caspase-1 activation and subsequent cleavage of effector proinflammatory cytokines including pro-IL-1*β* and pro-IL-18. 

Recently several studies highlighted with particular emphasis the relevance and the role of NLRP3 (previously known as CIAS1 and NLRP3) in the pathogenesis of CD [67, 68]. 

There is evidence suggesting that NLRP3 is able to respond to a variety of signals: adenosine triphosphate (ATP), nigericin, maitotoxin, *Staphylococcus aureus *and *Listeria monocytogenes *[69], and RNA and uric acid crystals (monosodium urate and calcium pyrophosphate dehydrate) released from dying cells [70, 71]. 

Literature data showed that the proinflammatory compound muramyl-dipeptide, the minimal bioactive peptidoglycan motif common to all bacteria, was an activator of the NLRP3 inflammasome, which suggested a very interesting connection between NOD2 and NALP3 [72]. 

On the other hand, Kanneganti et al. suggested that bacterial RNA and small antiviral compounds are the specific ligands of NLRP3 rather than MDP [70].

Anyway, NLRP3 plays a pivotal role in the inflammation regulating the activation of the caspase-1 and processing of IL-1*β*, two key mediators involved in the pathogenesis of the more common inflammatory disorders [73]. 

More recently, in an increasingly complicated picture, Elinav et al. described a novel regulatory sensing system in the colon, dependent on the NLRP6 inflammasome [74], and von Kampen et al. showed that CARD8 negatively regulates NOD-2 mediated signaling [75]. These current data further underline the link between the different components of the CD etiopathogenesis that are strongly correlated. 

## 3. Autophagy in CD

**Figure 5:**
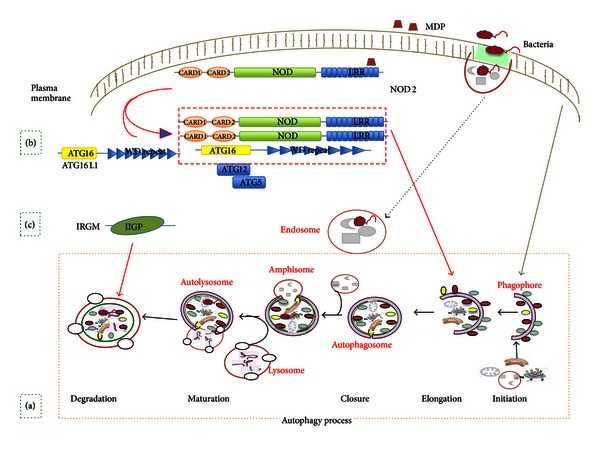
CD pathogenesis and autophagy: susceptible CD genes ATG16L1, NOD2, and IRGM are proteins critical for the autophagy process. (a) The process of mammalian autophagy is divided into the following principal steps: initiation, elongation, closure, maturation, and degradation. (b) At the bacterial entry site NOD2 activated by MDP recruit ATG16L1 to the plasma membrane. Follow the assembling of the ATG5-ATG12 complex, stabilized by ATG16L1, that facilitates the formation of an autophagosome around the invading bacterium. (c) IRGM, another autophagy-related gene, could be involved in the final steps of the degradation step.

Genomewide association studies and genetic analyses have emphasized the involvement of autophagy processes in the pathogenesis of inflammatory bowel diseases implicating three component genes in CD pathogenesis: ATG16L1 [76], IRGM [77], and NOD2 [78, 79]. These genes encode proteins critical for autophagy, a process that mediates degradation of intracellular proteins via vesicle-mediated delivery to the lysosome [80, 81]. Autophagy is involved in intracellular homeostasis, contributing to the degradation and recycling of cytosolic contents and organelles, as well as to resistance against infection and the removal of intracellular microbes. It is a major degradative pathway of the cell with several critical functions in innate and adaptive immunity [82] (Figure 5). 

The ATG16L1 deficiency mouse showed Paneth cell dysfunction with aberrant exocytosis, as well as an altered transcriptional profile, characterized by increased expression of pro-inflammatory cytokines and lipid metabolism genes like Paneth cell phenotype of CD patients [83]. Nevertheless an important observation derived from the ATG16L1 mouse model was that the murine norovirus infection, as well as the presence of the commensal bacteria, was required for the generation of these specific Paneth cell abnormalities [84]. ATG16L1 is essential for all forms of autophagy, and the coding mutation T300A is associated with increased risk of CD. Despite its ubiquitous expression, the defects associated with ATG16L1 polymorphisms have so far been described only within the gut, probably owing to the high microbial load in this tissue.

Nucleotide-binding-oligomerization-domain- (NOD-) like receptors (NLRs) represent ancient sentinels of the host innate immune system, and genetic variants in NLR genes are associated with complex chronic inflammatory barrier diseases [85]. The NOD2 gene is an intracellular sensor for the bacterial cell wall component muramyl-dipeptide, and loss-of-function variants in the human NOD2 gene have been associated with an increased susceptibility for CD [86, 87] in Caucasian populations of European ancestry [88], and particularly for ileal disease [89], and were found to be an important regulator of the commensal gut microbiota in mice [90]. NOD2 recognizes components of the bacterial cell wall and elicits an NF-*κ*B response and mediates the release of defensins, which are antimicrobial peptides. Evidence from MDP stimulation of NOD2-activated autophagy shows a link between genetic risk loci and highlights the importance of defining disease associated pathways and the potential of new roles for known genes [78]. Epithelial cells and dendritic cells containing Crohn's-disease-associated ATG16L1 and NOD2 variants show defects in antibacterial autophagy [79, 91]. In dendritic cells, these defects are associated with an impaired ability to present exogenous antigens to CD4+T cells [78]. A discussed model of Crohn's disease is the one in which individuals are genetically susceptible to a pathogen that triggers a compensatory and harmful immune response. Antibacterial autophagy, through ATG16L1, NOD2, and potentially other genes (IRGM), is consistent with this model. However, one of the most important experimental supports for this model comes from an unrelated study using *Citrobacter rodentium *to induce intestinal inflammation in NOD2  −/− mice [92]. These results illustrate a close relationship between NOD2, ATG16L1, and autophagy, affecting intracellular processing and communication with the adaptive immune system suggesting that genetic polymorphisms may affect both pathways concomitantly. 

IRGM belongs to the p47 Immunity-Related GTPase (IRG) family and is linked to CD by GWAS as a protein that is implicated in the autophagy mechanism [93]. The analysis of the interactions between 44 autophagy-associated human proteins and 83 viral proteins belonging to different RNA virus families revealed that IRGM was the autophagy-associated protein most targeted by these viruses. IRGM can interact with 12 viral proteins belonging to different viruses, such as HCV and HIV-1 [94, 95]. A recent study suggests that a polymorphism in IRGM could affect the binding and the consequent misregulation by a specific miRNA (miR-196) that is highly expressed in the intestinal tissue of patients with Crohn's disease. The consequence is that the xenophagy flux is not well regulated leading to the accumulation of bacteria in the lysosomal compartment. This study showed that greater IRGM expression leads to both colocalization of adherent invasive *E. coli *(AIEC) with the autophagy machinery and increased intracellular survival of the bacteria [96]. This and other strains of *E. coli *are more abundant in the mucosa of CD patients [90]. The ability of IRGM to induce autophagy and limit the replication of intracellular bacteria has been demonstrated with mycobacteria by inducing mitochondrial depolarization and can increase ROS production and cell death [97]. Finally IRGM could regulate inflammation by either regulating intracellular pathogens or cellular homeostasis much like ATG16L1.

These data provide further information and support for the hypothesis that microbial/viral interactions with the intestinal mucosa are required for disease generation and suggest that combinatorial models for CD pathogenesis are most relevant for the study of human disease pathogenesis. 

## 4. Concluding Remarks 

The diagnosis of CD is reached through the results of clinical, laboratory, radiographic, endoscopic, and histologic analyses. 

Radiological and endoscopic techniques are essential for the diagnosis of CD since its onset and are useful in assessing the inflammatory status of the intestinal mucosa. However, endoscopy is an invasive procedure. In children it can be traumatic and could have critical implications due to the more severe clinical manifestation and complication of the pediatric disease, that make the intestinal mucosal extremely thin and at risk of perforation. 

Noninvasive tests for CD already exist, including antibodies, imaging-based screens, and fecal biomarkers [98]. The specificity of existing methods ranges from 89% to 95% for CD and other inflammatory bowel diseases. However, these methods are limited to active disease and poorly sensitive (~55%). Their outcome can be confounded by other diseases, further limiting their clinical utility. Recently, high expectations are placed in diagnostic studies of the gastrointestinal microbiota, but further validations will be necessary before this tool is accepted in clinical practice [32, 99]. 

Research is moving forward in order to identify new and valid biomarkers for the diagnosis of the disease with the aim of replacing the use of invasive techniques. 

Currently, only the measurement of fecal calprotectin levels has achieved a place in clinical routine practice and is used as a marker for noninvasive determination of intestinal inflammation [99, 100]. This protein is an ideal marker because it is not degraded by the human microbiota. Levels of fecal calprotectin significantly increase in patients with CD, ulcerative colitis, infectious colitis, and, to a lesser extent, in tumors of the colon rectum, but not in patients with functional disorders, as in the case of the irritable bowel syndrome. In CD, the calprotectin assay reflects the activity of the disease, monitoring its progression, and can contribute to the decision about the correct medication strategy. 

The application of this test in the pediatric population is a good result. Although the test is not yet able to replace diagnostic colonoscopy, it can be a good indicator for the decision to use or delay the use of invasive investigations [99]. 

Recently, Vitali et al. suggested the use of high-mobility group box1 (HMGB1) as a novel marker of intestinal mucosal inflammation. HMGB1 is today regarded as a pleiotropic cytokine, that is passively released by necrotic cells, but not from apoptotic cells. Moreover HMGB1 could be actively secreted from some types of immune cells in response to lipopolysaccharide (LPS), IFN-*γ*, and TNF-*α*. There is evidence that HMGB1 is secreted in the stools of these patients and not detectable in controls [101]. 

The relationship between the genetic susceptibility and the microbiome could be considered in the disease diagnosis. There are several international human microbiome projects that have focused initially on the bacterial component of the microbiome. The evidence that bacteria play an important role in CD includes the observation that surgical diversion of the fecal stream ameliorates the inflammation and that antibiotics help some patients. Moreover other evidence is showed in mouse models of colitis where virus, bacteria, or both acting together can contribute to the pathology via signaling through innate immune sensors and regulation of pro- and anti-inflammatory cytokines [82]. The microbiome varies from person to person and such variation could provide environmental inputs that contribute to the incidence of CD, within the genetic foundation revealed by GWAS [102]. The concept of dysbiosis as a contributor to CD is correlated with intestinal bacteria communities, so the changes in the bacterial microbiota could have a potential role in the disease. However, this hypothesis needs to be expanded to include specific interactions between individual bacteria and host genes. 

Moreover, another ambitious goal is the identification of a genetic pattern able to associate specific phenotypic characteristics to CD patients or to anticipate the possible consequences of the disease already in childhood and thus prevent complications associated with the disease and to choose the best treatment for each patient. 

A rapid diagnosis is fundamental to avoid a growth delay or complications of the disease typical of the pediatric disease leading to surgery. In some cases, genetic studies have provided useful information for the identification of specific mutations that predict risk of stenosis and surgery and/or disease localization in pediatric-onset CD [103, 104]. 

The difficulty of finding a common genetic pattern of association is caused by the multifactorial feature of the disease that shows different characterizations by world region and race. 

In conclusion, it would certainly be useful to be able to create a biological algorithm that helps clinicians in the identification and classification of the disease and to determine the pharmacological care. This algorithm could include not only the known and principal factors predisposing to the disease, but also the gene-microbiome interaction and could help identify novel markers in patients with familiar history of EOCD. This could represent a major advance for early-onset diagnosis as specific tests might be developed to improve counselling, while direct identification of modifier genes might assist in the recognition of new genetic, environmental, and microbial causes of CD.
